# Advantages of Using Unweighted Approximation Error Measures for Model Fit Assessment

**DOI:** 10.1007/s11336-023-09909-6

**Published:** 2023-04-18

**Authors:** Dirk Lubbe

**Affiliations:** Section for Psychological Methods, Department of Psychology, Brandenburg Medical School Theodor Fontane, Am Alten Gymnasium 1-3, 16816 Neuruppin, Germany

**Keywords:** discrepancy function, goodness of fit, approximation error, structural equation model

## Abstract

Fit indices are highly frequently used for assessing the goodness of fit of latent variable models. Most prominent fit indices, such as the root-mean-square error of approximation (RMSEA) or the comparative fit index (CFI), are based on a noncentrality parameter estimate derived from the model fit statistic. While a noncentrality parameter estimate is well suited for quantifying the amount of systematic error, the complex weighting function involved in its calculation makes indices derived from it challenging to interpret. Moreover, noncentrality-parameter-based fit indices yield systematically different values, depending on the indicators’ level of measurement. For instance, RMSEA and CFI yield more favorable fit indices for models with categorical as compared to metric variables under otherwise identical conditions. In the present article, approaches for obtaining an approximation discrepancy estimate that is independent from any specific weighting function are considered. From these unweighted approximation error estimates, fit indices analogous to RMSEA and CFI are calculated and their finite sample properties are investigated using simulation studies. The results illustrate that the new fit indices consistently estimate their true value which, in contrast to other fit indices, is the same value for metric and categorical variables. Advantages with respect to interpretability are discussed and cutoff criteria for the new indices are considered.

**Supplementary Information:**

The online version contains supplementary material available at 10.1007/s11336-023-09909-6.

## Introduction

Assessing goodness of fit is an essential part of statistical modeling. Identifying systematic error and quantifying its size are important, because if a model does not fit well, parameter estimates may be considerably biased and, consequently, conclusions based on them may be erroneous (Browne and Cudeck, 1992; Bollen, 1989).

As is well known, systematic errors are common in parsimonious statistical models in the social, educational, and behavioral sciences. Consequently, model fit is frequently evaluated using fit indices, which help to assess the size of systematic errors and to classify whether the fit may be considered as good, acceptable, or poor. Examples for commonly used fit indices are the Root-Mean-Square Error of Approximation (Steiger and Lind, 1980)and the Comparative Fit Index(CFI, Bentler, 1990).

Most fit indices are defined through test statistics, whereby the latter are frequently assumed to follow either a central or a noncentral $\chi ^2$-distribution (Yuan, 2005). The assumption of a specific distribution is crucial for distinguishing between random (estimation) error and systematic (approximation) error (e.g., Steiger & Lind, 1980; Steiger et al., 1985). Specifically, estimation error is the random deviation of an estimate from its parameter, which depends on the specific sample. The approximation error is a fixed, nonstochastic quantity, which characterizes the systematic deviation between true parameters and their limiting approximation by the model.

If there is no systematic error, that is, if the model fits the data exactly, test statistic *T* is assumed to asymptotically follow a *central* $\chi ^2$-distribution, having an expected value equal to the model’s degrees of freedom ($\text {df}$). If there is systematic error, *T* is assumed to asymptotically follow a *noncentral* $\chi ^2$-distribution, with $E(T) = \text {df} + \lambda$, in which $\lambda$ is the noncentrality parameter. Because of the additive contribution of $\text {df}$ and $\lambda$ as well as the resulting increase by the latter in case of systematic error, the noncentrality parameter is a theoretically sound misfit quantity. A simple point estimate can be obtained as

1 $$\begin{aligned} {\hat{\lambda }} = T - \text {df}. \end{aligned}$$

While the noncentrality parameter is well suited for quantifying systematic error, its specific value is complex to interpret. For making sense of it, the specific manner in which *T* is obtained needs to be taken into account. More specifically, it needs to be considered what determines the scaling of *T* and, therefore, the scaling of $\lambda$.

In the context of covariance-structure models, *T* can be obtained using the normal theory maximum likelihood (ML) discrepancy (Jöreskog, 1969), or a (weighted) least-square discrepancy, that is

2 $$\begin{aligned} T_{LS} = n \cdot ({\varvec{s}} - \varvec{{\hat{\sigma }}})' {\varvec{W}} ({\varvec{s}} - \varvec{{\hat{\sigma }}}), \end{aligned}$$

in which $\varvec{{\hat{\sigma }}}$ is a vector of model implied covariances, ${\varvec{s}}$ is a vector of corresponding sample covariances, and ${\varvec{W}}$ is a weight matrix.

There are several candidates for ${\varvec{W}}$ for which *T* (depending on distributional assumptions) asymptotically follows a $\chi ^2$-distribution. Under normality assumptions, the generalized least squares (GLS) discrepancy can be used. It can be written as

$$\begin{aligned} {\varvec{W}} = 1/2 \cdot {\varvec{D}}'\left( {\varvec{S}}^{-1} \otimes {\varvec{S}}^{-1} \right) {\varvec{D}}, \end{aligned}$$

where ${\varvec{D}}$ is a duplication matrix (e.g., Browne & Arminger, 1995) and ${\varvec{S}}$ is the variables’ covariance matrix. This discrepancy function has the same solution as the ML discrepancy, but a different minimum. If instead the inverted asymptotic variance-covariance-matrix of the elements of ${\varvec{s}}$ is inserted for ${\varvec{W}}$, the asymptotically distribution free (ADF) discrepancy function is obtained (e.g., Browne, 1982). Other candidates for ${\varvec{W}}$, such as the identity matrix, which is used for obtaining the unweighted least squares (ULS) discrepancy, do usually not yield an approximately $\chi ^2$-distributed statistic.

If an appropriate weighting is selected, then the expected contributions of deviations per $\text {df}$ asymptotically follow mutually independent normal distributions having a variance of 1.0 and expectation $\mu _j$, with $j=1,\cdots ,\text {df}$. If there is no systematic difference between ${\varvec{s}}$ and $\varvec{{\hat{\sigma }}}$, the expected values of $\mu _j$ are 0.0. Accordingly, the square sum of expected values follows a central $\chi ^2$-distribution. In case of systematic error, that is, if $\mu _j \ne 0.0$, the square sum’s expected value is increased by $\lambda = \sum _j \mu _j^2$.

An important feature of this weighting is that specific values of $\mu _j$ directly depend on the standardization of variances of random errors. In other words, the scaling of $\lambda$ is determined by sampling properties. It follows that different $\lambda$-values are obtained depending on sample size (which can be easily controlled for) as well as the estimators’ variances.

However, why should the size of nonstochastic quantity $\lambda$ depend on the expected variance of random errors? In fact, relying on such a scaling entails potential limitations. These limitations relate to the (*i*) interpretability and (*ii*) universality of using $\lambda$ for quantifying systematic error.

For a simple illustration of the dependency between sampling properties and the size of $\lambda$, consider the following example. A model assumes a fixed correlation of zero, that is, ${\hat{\sigma }}=0.0$. The sample correlation’s expected value, however, is $E(s)=0.1$. The specific value of $\lambda$ (using Eq. (2)), depends on sample size *n* as well as the specific asymptotic variance of the sample estimate.

Consider that *n* is either 1000 or 10, 000, and that either the variables’ product-moment correlations (*pmc*) or the tetrachoric correlations (*tc*) are analyzed.^1^ The resulting estimates for ${\hat{\lambda }}$ are as follows: using *pmc* and $n=1000$, we obtain ${\hat{\lambda }} = 9.97$, and for $n=10,000$, ${\hat{\lambda }} = 99.97$. For *tc* we obtain ${\hat{\lambda }} = 4.11$ and ${\hat{\lambda }} = 41.09$ for $n=1000$ and $n=10,000$ respectively.

As is well known, the impact of differing sample size on the noncentrality parameter can be readily accounted for, dividing it by *n*. The RMSEA, which gives the square root of the average noncentrality parameter per observation and degree of freedom, may be written as

3 $$\begin{aligned} \text {RMSEA} = \sqrt{\textrm{max} \left( \frac{{\hat{\lambda }}}{n\cdot \text {df}},0\right) }. \end{aligned}$$

Accordingly, irrespective of sample size, RMSEA values based on product-moment correlations yield a value of roughly 0.1. However, for *tc*, which have larger variances, the RMSEA is 0.064. Note that widely accepted conventions consider RMSEA values smaller than 0.05 as indicating a good, values between 0.05 and 0.08 as an acceptable, and values larger than 0.10 as a poor model fit (e.g., Hu & Bentler, 1998). Thus, the fit of the *tc*-based model would be classified as acceptable and the fit of the *pmc*-based model as poor, although both models approximate the same structure.

Clearly, systematic variance differences between types of correlation coefficients are not accounted for by the RMSEA—nor by any other noncentrality parameter based fit index. This feature of fit indices, particularly their dependence on the variables’ level-of-measurement, has been reported by several authors (e.g., Maydeu-Olivares & Joe, 2014; Monroe & Cai, 2015; Xia & Yang, 2018; Savalei, 2021). Specifically, previous research indicates that common fit indices, such as RMSEA, CFI and TLI (Tucker-Lewis Index), generally yield overly optimistic fit assessments when analyzing categorical as compared to metric variables.

Obviously, the source of the systematic difference in fit indices is the weighting of discrepancies, which depends on the variances of the respective type of estimate. Thus, any approach for resolving the outlined limitation would need to address the impact of the random-error-related weighting.

A previous approach that resolves one of the two limiting aspects of this weighting has been proposed by Savalei (2021). While the general impact of random-error-related scaling is left unaltered, the difference in fit indices for metric and categorical data analyses is controlled for. Specifically, this adjustment rescales RMSEA-values pertaining to categorical data models in a way, that they approximate the expected value that would have been obtained analyzing metric data. This approach has the advantage that conventional cut-off criteria for the RMSEA can be used for categorical data as well. However, the scaling related attributes with respect to the interpretability of specific values are not addresses in this way.

Another approach, which is pursued in more detail in the following, aims to eliminate the impact of random-error-related scaling entirely. Fit indices that yield an unweighted discrepancy measure are already available. Examples are the standardized root-mean-square residual (SRMR, Jöreskog & Sörbom, 1988) and the correlation root-mean-squared residual (CRMR, Bollen, 1989). However, these measures do not distinguish between random and systematic error and, therefore, overestimate their population parameters. Fortunately, this limitation has been addressed by Maydeu-Olivares (2017), who proposed an estimation approach for the population parameters of SRMR and CRMR. This proposal will be revisited below. A remaining limiting property of these indices, however, is that they merely address average discrepancies irrespective the model’s relative complexity.

The present article proposes a more general approach than the previous fit index adjustments. Specifically, it considers ways in which a “general-purpose” unweighted approximation error estimate can be obtained. Based on this estimate, different specific fit indices can be calculated. In contrast to Savalei (2021), the new indices fully eliminate the random-error-related scaling, while similarly maintaining the equality of fit indices for metric and categorical data models. Consequently, by eliminating the specific scaling, individual fit index values have a comparably simple interpretation that can be directly linked to the size of covariance residuals. In contrast to the population estimates of SRMR and CRMR, as proposed by Maydeu-Olivares (2017), fit indices that contain an unweighted approximation error offer more differentiated options for fit assessment. Particularly, such indices can (a) be implemented into fit indices that take the model’s complexity into account and (b) they can be used to assess *absolute* as well as *relative* fit. Specifically, absolute fit can be assessed by modifying the RMSEA with the new approximation error estimate, and relative fit can be considered by modifying the CFI.

## Unweighted Approximation Error Estimate

The unweighted approximation error $\lambda _\text {u}$ can be defined as the sum of squared discrepancies due to approximation errors. It may be expressed as the population value of the unweighted least square (ULS) discrepancy, that is

4 $$\begin{aligned} \lambda _\text {u} = n \cdot (\varvec{\sigma }_0 - \varvec{\sigma })' (\varvec{\sigma }_0 - \varvec{\sigma }), \end{aligned}$$

in which $\varvec{\sigma }_0$ and $\varvec{\sigma }$ are the corresponding population covariances of ${\varvec{s}}$ and $\varvec{{\hat{\sigma }}}$ respectively. Note that the ULS discrepancy is merely a means to expressing the unweighted approximation error. It is not involved in model fitting, which can be performed by an entirely different function.

It is already known from other standardized fit indices, such as SRMR and CRMR, that it is only sensible to report a unit-weighted fit measure, if variables are scaled identically. Thus, the following considerations assume that a model’s correlation structure is used for calculating $\lambda _\text {u}$. Consequently, the unweighted approximation error equals *n*-times the squared deviations between the true population correlations and their limiting approximation by the model. Note that, similar to SRMR and CRMR, model estimation does not necessarily need to be based on sample correlations.

Although $\lambda _\text {u}$ may be considered as a noncentrality parameter pertaining to the ULS discrepancy, it cannot be simply approximated using Eq. (1). Instead, there are two other general approaches in which a suitable estimate may be obtained. First, an adjusted estimate for the degrees of freedom $\text {df}$ can be calculated, which can be inserted into (1). Second, a scaling constant that accounts for the relative amount of systematic error in relation to overall error can be used to rescale the ULS sample discrepancy.

For implementing the first general approach, there are different options. For one, standard theory in connection with robust adjustments of test statistics based on oversimplified least-square estimation can be used as rationale (e.g., Muthen, 1997). Also, the upper mentioned approach of (Maydeu-Olivares, 2017) can be adapted. While both options are closely related, they have few distinct features.

**Approach (1.1).** In connection with ULS estimation, there are robust corrections available, which can be used for obtaining a model test statistic that approximates the expected value and variance of the $\chi ^2$-distribution for the respective $\text {df}$ of the model ( e.g., Satorra, 1992). While this approach is commonly used for correcting model test statistics, it can also be used to approximate Eq. (4).

A mean-variance adjusted ULS test statistic (usually abbreviated as ULSMV) can be obtained based on an estimate of the correlation estimates’ covariance matrix $\varvec{\Gamma }$ and the Jacobian matrix containing the derivatives of model implied correlations with respect to the model parameters $\varvec{\Delta }$. A more detailed description of these matrices in connection with robust adjustments can be found in Muthen (1997).

For ULS, the adjusted test statistic is calculated as

$$\begin{aligned} T_{adj} = \frac{T_\text {ULS}}{a}, \end{aligned}$$

in which

$$\begin{aligned} a = \text {tr}\left[ {\varvec{U}}_{(1.1)} \varvec{\Gamma } \right] /d^*, \end{aligned}$$

with

$$\begin{aligned} {\varvec{U}}_{(1.1)} = {\varvec{I}} - \varvec{\Delta } \left( \varvec{\Delta }' \varvec{\Delta } \right) ^{-1} \varvec{\Delta }', \end{aligned}$$

and

$$\begin{aligned} d^* = \frac{\text {tr}\left[ {\varvec{U}}_{(1.1)} \varvec{\Gamma } \right] ^2}{\text {tr}\left[ ({\varvec{U}}_{(1.1)} \varvec{\Gamma })^2 \right] }. \end{aligned}$$

From the adjusted test statistic $T_{adj}$ and degrees of freedom $d^*$ a noncentrality parameter estimate can be obtained by inserting these values into Eq. (1). The unweighted discrepancy function can then be obtained by rescaling the value using *a*. Simplifying the resulting expression yields

5 $$\begin{aligned} {\hat{\lambda }}_{\text {u}(1.1)} = T_\text {ULS} - \text {tr}\left[ {\varvec{U}}_{(1.1)} \varvec{\Gamma } \right] . \end{aligned}$$

**Approach (1.2).** Another unweighted approximation error estimate can be obtained based on a minor reformulation of the approach of Maydeu-Olivares (2017), which was originally designed to approximate the population value of SRMR and CRMR. Specifically, the noncentrality parameter estimate can be calculated as

6 $$\begin{aligned} {\hat{\lambda }}_{\text {u}(1.2)} = T_\text {ULS} - n \cdot \text {tr}\left( \varvec{{\hat{\Sigma }}_e} \right) , \end{aligned}$$

in which $\varvec{{\hat{\Sigma }}_e}$ is the variance-covariance matrix of the (correlation) residuals, that is

7 $$\begin{aligned} \varvec{{\hat{\Sigma }}_e} = {\varvec{U}}_{(1.2)} \varvec{\Gamma } {\varvec{U}}_{(1.2)}', \end{aligned}$$

with

$$\begin{aligned} {\varvec{U}}_{(1.2)} = {\varvec{I}} - \varvec{\Delta }\left( \varvec{\Delta ' W \Delta }\right) ^{-1} \varvec{\Delta ' W}, \end{aligned}$$

in which ${\varvec{W}}$ is the weight matrix of the GLS discrepancy function and $\varvec{\Gamma }$ as well as $\varvec{\Delta }$ are defined as above.

Clearly, approaches (1.1) and (1.2) are closely related. The central difference is that approach (1.2) additionally includes ${\varvec{W}}$. While this is important for estimating the correlations’ variance-covariance matrix, the immediate impact for obtaining an appropriate estimate of $\lambda _\text {u}$ is not obvious.

Approaches (1.1) and (1.2) solely depend on (*i*) the sample correlation, (*ii*) an estimation of their variance-covariance matrix, and (*iii*) the parameter estimates of a model. The specific discrepancy function used for fitting the model’s parameter estimates is only relevant for (*iii*). However, because of the equivalence between ML and GLS, approach (1.2) might be particularly suited in connection with ML parameter estimates.

**Approach (2).** The second approach is based on the assumption that (*i*) sources of random and systematic error are independent and that (*ii*) the test statistic’s expected value results from the additive contribution of $\lambda$ and $\text {df}$. If both assumptions are satisfied, then the corresponding test statistic asymptotically follows a (non-)central $\chi ^2$-distribution. It then follows that the relative proportion of variances attributed to systematic error in relation to the total error can be expressed as

8 $$\begin{aligned} \widehat{\text {ADR}} = \frac{{\hat{\lambda }}}{T}, \end{aligned}$$

which is henceforth referred to as Approximation Discrepancy Ratio (ADR). The ML discrepancy function is a viable candidate for calculating ADR, because it yields a close approximation of the noncentral $\chi ^2$-distribution under various misspecification scenarios (e.g., Curranet al., 2002). However, while any discrepancy function yielding the above properties is suited for obtaining *T*, in practice, the specific choice might have a considerable impact on the results, because of their notable performance differences (e.g., Olsson et al., 2004; Shi & Maydeu-Olivares, 2020)

The result of (8) can then be used to weigh test statistic $T_\text {ULS}$, that is

9 $$\begin{aligned} {\hat{\lambda }}_{\text {u}(2)} = \widehat{\text {ADR}} \cdot T_\text {ULS}, \end{aligned}$$

which yields the proportion of $T_\text {ULS}$ that is attributed to systematic error. It is important to notice, that $T_\text {ULS}$ and *T* (used in the calculation of ADR) originate from different discrepancy functions. In this way, the desired scaling of $T_\text {ULS}$ is combined with an appropriate measure of the relative contribution of systematic error. It is easy to see that, if the same discrepancy function would be used for both quantities, the trivial result ${\hat{\lambda }} = {\hat{\lambda }}/T \cdot T$ would be obtained.

The second approach has the advantage that it is (*a*) computationally rather simple and (*b*) that the result of any discrepancy function can be used without requiring any specific adaption, as long as the corresponding distributional assumptions are satisfied. However, the inclusion of the results of a second discrepancy function make this approach conceptually more complex and it is difficult to say under which conditions the upper mentioned assumptions are met.

Considering property (*b*), approach (2) is similarly applicable to metric as well as categorical data. In order to apply approaches (1.1) and (1.2) to categorical data models (for instance based on the variables’ polychoric correlations), matrices $\varvec{\Gamma }$, $\varvec{\Delta }$, and ${\varvec{W}}$ need to be selected accordingly.

Another interesting feature of approach (2) is that it can be calculated retrospectively without having the original data, as long as the model fit test statistic as well as the square sum of correlation residuals are available. The size of the latter may also be inferred from the CRMR.

Although approaches (1) and (2) proceed differently, there is one direct connection. If approach (2) is used in connection with ULSMV estimation, its yields the same result as approach (1.1).

### New Fit Indices

Based on the unweighted approximation error, new fit indices can be calculated by substituting the noncentrality parameter in their well-established counterparts. An unweighted Root-Mean-Square Error of Approximation RMSEA$_u$ can be calculated analogous to equation (3), yielding

10 $$\begin{aligned} \text {RMSEA}_u = \sqrt{\text {max} \left( \frac{{\hat{\lambda }}_\text {u}}{n \cdot \text {df}}, 0 \right) }. \end{aligned}$$

For correlation structure models, RMSEA$_u$ yields the average absolute correlation residual due to approximation error per $\text {df}$. For instance, RMSEA$_u=0.05$ denotes that the average absolute correlation residual per $\text {df}$ due to systematic error is 0.05. Clearly, this interpretation is rather simple and intuitive as compared to that of the original RMSEA, for which the scaling of the noncentrality parameter needs to be considered.

In order to obtain an asymptotically unbiased estimate for the population value of equation (10), minor adjustments are required. For approaches (1.1) and (1.2) a correction constant can be obtained as follows, which is derived using Taylor expansions of moments of functions of random variables, which is outlined in more detail for approach (2).

For approach (1.2) Maydeu-Olivares (2017) has given the following adjustment. For a simplified notation, let

$$\begin{aligned} F_\text {ULS} = T_\text {ULS}/n \end{aligned}$$

Then, the estimate of Eq. (10) based on approach (1.2) is

$$\begin{aligned} \text {RMSEA}_{u(1.2)} = \frac{1}{k_{(1.2)}} \sqrt{\text {max} \left( \frac{F_\text {ULS}-\text {tr} \left( \varvec{{\hat{\Sigma }}_e} \right) }{\text {df}}, 0 \right) } \end{aligned}$$

in which *k* is the correction constant, that is

$$\begin{aligned} k_{(1.2)} = 1 - \frac{\sigma ^2_F}{4 \cdot F_\text {ULS}^2}, \end{aligned}$$

with

$$\begin{aligned} \sigma ^2_F = 2 \cdot \text {tr}(n^{-1} \varvec{{\hat{\Sigma }}_e}) + 4 \cdot ({\varvec{s}} - \varvec{{\hat{\sigma }}})' (n^{-1} \varvec{{\hat{\Sigma }}_e}) ({\varvec{s}} - \varvec{{\hat{\sigma }}}) \end{aligned}$$

The same approach can be used for approach (1.1), replacing $\varvec{{\hat{\Sigma }}_e}$ with $n^{-1} \text {tr}\left[ {\varvec{U}}_{(1.1)} \varvec{\Gamma } \right]$.

For approach (2), a bias corrected RMSEA$_u$ estimate can be obtained using

$$\begin{aligned} \text {RMSEA}_{u(2)} = \frac{1}{k_{(2)}} \sqrt{\text {max} \left( \frac{\text {ADR} \cdot F_\text {ULS}}{\text {df}}, 0 \right) }, \end{aligned}$$

with

$$\begin{aligned} k_{(2)} = 1 - \frac{\sigma ^2_F}{8 \cdot F_\text {ULS}^2}. \end{aligned}$$

The correction factor as well as standard errors of $f(F_\text {ULS}) = \text {RMSEA}_{u(2)}$ can also be estimated using the Delta method (e.g., Wolter, 1985). Specifically, expected value and variance are

$$\begin{aligned} \begin{aligned} E\left[ f(F_\text {ULS})\right]&= f(F_\text {ULS}) + \frac{f''(F_\text {ULS})}{2} \sigma ^2_F \\&= \sqrt{\frac{\textrm{ADR} \cdot F_\text {ULS}}{d}} - \frac{1}{8} \sqrt{\frac{\textrm{ADR}}{F_\text {ULS}^3 \cdot d}} \sigma ^2_F \\&= \sqrt{\frac{\textrm{ADR} \cdot F_\text {ULS}}{d}} \left( 1 - \frac{\sigma ^2_F}{8 \cdot F_\text {ULS}^2}\right) \end{aligned} \end{aligned}$$

and

$$\begin{aligned} \begin{aligned} \textrm{Var}\left[ f(F_\text {ULS})\right]&= \left[ f'(F_\text {ULS})\right] ^2 \sigma ^2_F \\&= \frac{\sigma ^2_F \cdot \textrm{ADR}}{4 \cdot F_\text {ULS} \cdot d}, \end{aligned} \end{aligned}$$

given that

$$\begin{aligned} f'(F_\text {ULS}) = \sqrt{\frac{\textrm{ADR}}{4 \cdot F_\text {ULS} \cdot d}} \end{aligned}$$

and

$$\begin{aligned} f''(F_\text {ULS}) = -\sqrt{\frac{\textrm{ADR}}{16 \cdot F_\text {ULS}^3\cdot d}} \end{aligned}$$

RMSEA values are commonly reported together with their $90\%$ confidence interval. Assuming that the sampling distribution of the indices approximately follows a normal distribution in large samples, the interval may be calculated as

$$\begin{aligned} \textrm{RMSEA}_u \pm z_{1-\alpha /2} \cdot se, \end{aligned}$$

in which *se* is the asymptotic standard error of $RMSEA_u$.

For approach (1.2), the asymptotic standard error proposed by Maydeu-Olivares (2017) can be used with one minor modification, replacing the number of nonredundant covariances/correlation with $\text {df}$. This yields

$$\begin{aligned} se_{(1)} = \sqrt{\frac{\sigma ^2_F}{k^2_{(1)} \cdot 4 \cdot \text {df} \cdot F_\text {ULS}}}. \end{aligned}$$

For approach (2), a similar approach can be used. Carrying the $1/k_{(2)}$ scaling forward, the asymptotic standard error is otherwise identical to the square root of expression of $\textrm{Var}\left[ f(F_\text {ULS})\right]$, given above, yielding

$$\begin{aligned} se_{(2)} = \sqrt{\frac{\sigma ^2_F \cdot \textrm{ADR}}{k_{(2)}^2 \cdot 4 \cdot F_\text {ULS} \cdot \text {df}}}. \end{aligned}$$

An unweighted version of the Comparative Fit Index (CFI) can be obtained in a similar manner. The CFI considers the relation between the noncentrality parameter of the fitted model to that of a less complex base model (usually an independence model). Again, the noncentrality parameter is replaced by ${\hat{\lambda }}_\text {u}$. Specifically, the CFI$_u$ may be written as

11 $$\begin{aligned} \text {CFI}_u = 1 - \frac{\text {max} \left( {\hat{\lambda }}_\text {u}, 0 \right) }{\text {max} \left( {\hat{\lambda }}_\text {u}, {\hat{\lambda }}_{B \cdot \text {u}} \right) }, \end{aligned}$$

in which ${\hat{\lambda }}_{B \cdot \text {u}}$ is the unweighted approximation error estimate for the base model. Because estimates of $\lambda _\text {u}$ are not systematically biased, no additional adjustments are required.

The interpretation of CFI$_u$ may also be considered as somewhat simpler than that pertaining to the original CFI. Specifically, CFI$_u$ expresses the size of systematic error variance of the base model relative to that of the fitted model. For instance, CFI$_u = 0.90$ denotes that the squared correlation residuals of a specific model are $90\%$ smaller than those of the baseline model.

The present considerations are restricted to RMSEA and CFI, because they are prototypical indices which include a noncentrality parameter estimate in their original formulation. Other relative fit indices that use a similar expression as the CFI, such as the Tucker-Lewis Index (TLI) or Normed Fit Index (NFI), lack this property and are, therefore, not suitable for the current purpose.

While specific values of RMSEA$_u$ and CFI$_u$ have a simple interpretation that can be directly connected to the absolute or squared correlation residuals, it is not obvious which values might indicate a good, acceptable or poor fit. There are two option for identifying suitable cutoff values for the new indices. First, by expert consensus based on empirical examples, which is the approach with which cutoff values for most fit indices have been established (e.g., Hu & Bentler, 1998). Second, by comparing established and new indices. If they are in a relatively simple relation, this may provide sufficient information to establish suitable cutoffs. While the first approach is beyond the scope of this article, the second approach will be pursued in the next section using simulation studies.

To assist with the calculation of the new indices, the supplementary materials to this article contain a data example (’example_data.txt’) as well as the corresponding R-code (’R_example.pdf’) for calculating RMSEA$_u$ and CFI$_u$ based on approaches (1.1), (1.2), and (2). The data is metric and contains 4 variables and 1000 cases. The corresponding analysis assumes one latent variable. The R-code can be also used for evaluating other data sets and models. For more information, see the instructions in the materials.

## Simulation Study

For investigating the properties of the new indices, a simulation study was conducted considering a set of varying confirmatory factor model structures. These simulations pursue the goals to demonstrate (*i*) that accurate estimates of $\lambda _\text {u}$ can be obtained in finite samples, (*ii*) that fit indices based on the new measure yield identical values for models based on metric and dichotomous variables for identical model structures, and (*iii*) to identify suitable cutoff values for RMSEA$_u$ and CFI$_u$ by comparing them to their noncentrality-parameter-based counterparts.

### Method

Data were generated based on population correlation matrices $\varvec{\Sigma }_0$ for a given *q*-factorial model structure. These matrices were calculated from predefined $p \times q$ matrices of item loadings ${\varvec{K}}$, a $q \times q$ matrix $\varvec{\Phi }$ of factor inter-correlations, and a $p \times p$ diagonal matrix $\varvec{\Psi }$ of residuals, that is

$$\begin{aligned} \varvec{\Sigma }_0 = {\varvec{K}} \varvec{\Phi } {\varvec{K}}' + \varvec{\Psi }. \end{aligned}$$

Factor loadings were chosen in a way that population correlation matrix $\varvec{\Sigma }_0$ yielded a predefined ULS-discrepancy from the best approximating correlation matrix implied by the model. Specifically, loadings were chosen in a way that the true approximation discrepancies yielded predefined target values for each of the simulation settings. This was achieved by numerical optimization, iteratively optimizing the population model discrepancy with respect to the fit indices’ target values based on the model parameters using the Broyden–Fletcher–Goldfarb–Shanno (BFGS) algorithm as implemented in the *optim*-procedure in R. Two target value settings were considered: (*i*) RMSEA$_u=0.05$; CFI$_u=0.95$ and (*ii*) RMSEA$_u=0.05$; CFI$_u=0.99$.

The source of systematic error was the $q^{th}$ latent variable, which, in addition to the first $q-1$ latent variables, was considered for generating the data, whereas the analysis model only considered the structure of the first $q-1$ latent variables. For introducing additional variability to the way in which systematic misfit was generated, two different types of loading patterns were used for the $q^{th}$ latent variable.

For the first type, the $q^{th}$ latent variable had loadings on the first and last indicator pertaining to each of the prior $q^*=q-1$ latent variables. For instance, for a three-factorial model structure with overall $p=8$ variables, the loading matrix would be

$$\begin{aligned} {\varvec{K}} = \begin{pmatrix} k_{11} &{}k_{21}&{}k_{31}&{}k_{41} &{}0 &{}0 &{}0 &{} 0 \\ 0 &{}0 &{}0 &{}0 &{}k_{52} &{}k_{62}&{}k_{72}&{}k_{82} \\ k^*_{13}&{}0 &{}0 &{}k^*_{43}&{}k^*_{53}&{}0 &{}0 &{}k^*_{83} \end{pmatrix}'. \end{aligned}$$

For the second type, the $q^{th}$ latent variable had positive loadings on the first half of the indicators pertaining to each of the prior $q^*$ latent variables, and negative loadings on the second half. Consequently, the three-factorial structure with $p=8$ variables would be

$$\begin{aligned} {\varvec{K}} = \begin{pmatrix} k_{11} &{}k_{21} &{}k_{31} &{}k_{41} &{}0 &{}0 &{}0 &{} 0 \\ 0 &{}0 &{}0 &{}0 &{}k_{52} &{}k_{62} &{}k_{27} &{}k_{82} \\ k^*_{13}&{}k^*_{23}&{}-k^*_{33}&{}-k^*_{43}&{}k^*_{53}&{}k^*_{63}&{}-k^*_{73}&{}-k^*_{83} \end{pmatrix}'. \end{aligned}$$

The two loading patterns are henceforth referred to as misfit type I and misfit type II.

For the first $q^*$ latent variables, loadings adhered to a simple structure, that is, each variable had loadings on one latent variable only. For simplicity, loadings on the first $q^*$ latent variables had identical loadings *k*. Similarly, loadings on the $q^{th}$ latent variable had identical values $k^*$. Correlations between the first $q^*$ factors were set to 0.3 by default. The $q^{th}$ latent variable was assumed independent from the remaining $q-1$ latent variables.

Altogether, 56 model settings were considered. In addition to misfit type and target values for RMSEA$_u$ and CFI$_u$, the number of variables *p*, the number of latent variables $q^*$, and sample size *n* were varied. Specifically, the combinations of $p=8$, 12, and 18 and $q^*=1$, 2 and 3 were considered, omitting the combinations of $q^*=1$ and $p=18$ as well as $q^*=3$ and $p=8$. Sample sizes were $n=250$ and 1000. The specific values for *k* and $k^*$ for the different model settings, rounded to three decimal places, are given in Table 1.

**Table 1:** True loadings for the population models for the different model settings.

$q^*$	*p*		Misfit type I						Misfit type II				
			CFI$_u=.95$			CFI$_u=.99$			CFI$_u=.95$			CFI$_u=.99$	
			*k*	$k^*$		*k*	$k^*$		*k*	$k^*$		*k*	$k^*$
1	8		.588	.216		.883	.216		.339	.760		.866	.412
	12		.584	.211		.874	.211		.423	.529		.840	.379
2	8		.537	.217		.807	.216		.519	.525		.797	.517
	12		.532	.213		.798	.213		.312	.758		.780	.431
	18		.521	.205		.777	.205		.413	.485		.743	.384
3	12		.449	.213		.674	.213		.435	.643		.666	.638
	18		.436	.207		.653	.207		.415	.524		.640	.516

For each of the 56 settings, 1000 data sets were sampled from a multivariate normal distribution based on corresponding correlation matrices $\varvec{\Sigma }_0$. These correlation matrices were then analyzed using confirmatory factor analyses based on ML and ULSMV estimation. For comparing fit indices between metric and dichotomous variables, additional dichotomized data sets were generated from the existing data by splitting metric variables at their mean. The dichotomized data were analyzed based on their tetrachoric correlation matrices using ULSMV.^2^

For the analyses based on ML estimation, all approximation error estimates were calculated using approach (1.1), (1.2), and (2). For the ULSMV analyses, only approaches (1.1) and (1.2) were included, because of the equivalence of (1.1) and (2) under this specific condition. Finally, RMSEA$_u$ and CFI$_u$ based on the different approaches, as well as their noncentrality-parameter-based counterparts were calculated for each model and averaged within simulation settings.

#### Results

Results pertaining to models using ML and ULSMV estimation are presented separately. First, the results of metric data analyses with ML estimation are considered. Tables 2 and 3 give RMSEA$_u$ estimates as well as the coverage probabilities of the corresponding $90\%$-confidence intervals based on the different approaches for misfit type I and II respectively. Clearly, the average estimates of RMSEA$_u$ are very close to their target values of 0.05 irrespective of the estimation approach. This holds true across all simulation settings, implying that the unweighted approximation error estimate performs well if calculated based on multivariate normal indicators and the ML test statistic. For sample sizes of $n=1000$ deviations of the average estimate from the target value are seldom larger than $\pm 0.001$.

While neither type of misfit, nor the complexity of the model appears to have a distinct impact on the accuracy of average values, approach (2) has a tendency to overestimate the true RMSEA$_u$ value in conditions in which the true CFI$_u$ value is 0.99. These are also the conditions, in which average loadings are distinctly higher than for CFI$_u=0.95$ (see also Table 1). In such conditions, in which reproduced correlations are large, absolute deviations between sample and model implied correlations are more strongly weighted by typical discrepancy functions. Accordingly, because ADR-estimates are obtained based on the ML discrepancy function, this might have caused this slight overestimation.

Comparing values of the original RMSEA, it becomes obvious that they do not directly compare to RMSEA$_u$. For more complex models, as well as higher loadings, typical RMSEA values clearly exceed RMSEA$_u$ values. Particularly, in simulation settings with CFI$_u=0.99$, this trend can be observed. The reason for this is obvious. Because the elements of $\varvec{\Sigma }_0$ are comparably more extreme than in these settings, standard errors are smaller, and the scaling of the corresponding (weighted) discrepancy is relatively increased. Thus, the noncentrality parameter as well as the RMSEA are increased accordingly.

**Table 2:** Average RMSEA$_u$ and RMSEA values as well as $90\%$-CI coverages of ML confirmatory factor models based on 1000 replications per cell. (Misfit Type I).

Target	$q^*$	*p*	*n*		Appr. (1.1)			Appr. (1.2)			Appr. (2)			Orig.
value					Est.	CIC		Est.	CIC		Est.	CIC		Est.
RMSEA$_u=.05$; CFI$_u=.95$	1	8	250		.048	.822		.046	.827		.047	.674		.052
			1000		.050	.885		.050	.886		.049	.824		.056
		12	250		.049	.817		.047	.830		.045	.678		.053
			1000		.050	.854		.050	.856		.048	.804		.053
	2	8	250		.049	.820		.046	.829		.050	.726		.057
			1000		.050	.902		.050	.904		.050	.878		.061
		12	250		.050	.817		.048	.829		.049	.746		.058
			1000		.050	.890		.050	.899		.050	.870		.059
		18	250		.050	.801		.047	.822		.047	.718		.054
			1000		.050	.863		.050	.873		.049	.824		.054
	3	12	250		.050	.822		.047	.837		.051	.791		.062
			1000		.050	.887		.050	.893		.051	.875		.063
		18	250		.051	.784		.048	.821		.050	.762		.059
			1000		.050	.879		.050	.890		.050	.870		.059
RMSEA$_u=.05$; CFI$_u=.99$	1	8	250		.050	.849		.048	.863		.047	.760		.075
			1000		.050	.887		.050	.891		.049	.855		.077
		12	250		.050	.839		.048	.859		.046	.744		.074
			1000		.050	.858		.050	.863		.049	.830		.074
	2	8	250		.052	.892		.049	.906		.056	.878		.104
			1000		.050	.918		.050	.925		.052	.909		.106
		12	250		.051	.861		.049	.880		.054	.875		.104
			1000		.050	.908		.050	.921		.051	.899		.104
		18	250		.051	.833		.048	.852		.052	.888		.095
			1000		.050	.867		.050	.880		.051	.875		.095
	3	12	250		.051	.861		.048	.883		.056	.855		.146
			1000		.051	.889		.050	.908		.052	.878		.147
		18	250		.052	.826		.048	.874		.054	.849		.136
			1000		.050	.895		.050	.908		.051	.913		.136

Est.: RMSEA estimate and CIC: $90\%$-confidence interval coverage pertaining to the respective approach; Orig. Est.: unadjusted RMSEA.

The coverage probabilities of the $90\%$-confidence interval (CIC) are adequate. Particularly, if $n=1000$, results are close to $90\%$. For smaller sample sizes, CICs remain somewhat below their target. Also, confidence intervals coverages pertaining to approaches (1.1) and (1.2) are somewhat closer to $90\%$ than those of approach (2). For bias type II and CFI$_u=0.99$, all confidence intervals are somewhat too wide for $n=1000$, irrespective of the condition.

**Table 3:** Average RMSEA$_u$ and RMSEA values as well as $90\%$-CI coverages of ML confirmatory factor models based on 1000 replications per cell. (Misfit Type II).

Target	$q^*$	*p*	*n*		Appr. (1.1)			Appr. (1.2)			Appr. (2)			Orig.
value					Est.	CIC		Est.	CIC		Est.	CIC		Est.
RMSEA$_u=.05$; CFI$_u=.95$	1	8	250		.049	.844		.048	.851		.050	.756		.059
			1000		.050	.897		.050	.901		.050	.860		.061
		12	250		.050	.875		.049	.885		.052	.806		.068
			1000		.050	.929		.050	.932		.051	.906		.068
	2	8	250		.049	.827		.046	.833		.048	.691		.050
			1000		.050	.900		.050	.906		.049	.840		.053
		12	250		.050	.826		.047	.849		.049	.746		.057
			1000		.050	.895		.050	.904		.050	.865		.058
		18	250		.050	.808		.047	.831		.050	.799		.060
			1000		.050	.877		.050	.898		.050	.868		.060
	3	12	250		.051	.847		.048	.866		.047	.730		.054
			1000		.051	.905		.050	.913		.049	.858		.054
		18	250		.051	.790		.047	.824		.049	.777		.058
			1000		.051	.881		.050	.900		.050	.875		.057
RMSEA$_u=.05$; CFI$_u=.99$	1	8	250		.050	.890		.048	.904		.051	.862		.091
			1000		.050	.920		.050	.923		.050	.910		.092
		12	250		.050	.919		.048	.927		.053	.906		.113
			1000		.050	.954		.050	.959		.051	.936		.112
	2	8	250		.053	.871		.048	.900		.053	.879		.086
			1000		.051	.922		.050	.927		.051	.925		.087
		12	250		.052	.888		.048	.903		.054	.914		.105
			1000		.051	.929		.050	.943		.051	.936		.105
		18	250		.051	.869		.046	.881		.056	.908		.124
			1000		.051	.956		.049	.964		.052	.940		.124
	3	12	250		.053	.881		.047	.893		.054	.953		.109
			1000		.051	.932		.050	.949		.051	.940		.109
		18	250		.053	.858		.045	.865		.055	.966		.132
			1000		.051	.948		.049	.951		.052	.966		.132

Est.: RMSEA estimate and CIC: $90\%$-confidence interval coverage pertaining to the respective approach; Orig. Est.: unadjusted RMSEA.

Table 4 gives the CFI$_u$ estimates based on the different approaches for the metric data analyses with ML estimation. Similar to the RMSEA$_u$ estimates, values are consistently close to their target value.

**Table 4:** Average CFI $_u$ and CFI values of ML confirmatory factor models based on 1000 replications per cell.

Target	$q^*$	*p*	*n*		Misfit type I					Misfit type II			
value					(1.1)	(1.2)	(2)	Orig.		(1.1)	(1.2)	(2)	Orig.
CFI$_u=.95$	1	8	250		.954	.950	.952	.904		.952	.944	.950	.889
			1000		.950	.951	.950	.904		.951	.949	.951	.889
		12	250		.953	.951	.951	.888		.952	.938	.949	.832
			1000		.951	.952	.951	.891		.950	.947	.950	.835
	2	8	250		.955	.944	.951	.906		.956	.954	.953	.927
			1000		.951	.948	.950	.904		.952	.953	.952	.927
		12	250		.954	.946	.950	.891		.955	.947	.950	.893
			1000		.951	.949	.950	.892		.951	.949	.950	.894
		18	250		.954	.950	.950	.882		.955	.944	.950	.858
			1000		.950	.951	.950	.884		.951	.949	.950	.862
	3	12	250		.955	.944	.950	.888		.954	.950	.948	.908
			1000		.951	.948	.950	.888		.951	.951	.949	.911
		18	250		.952	.944	.947	.875		.954	.947	.948	.878
			1000		.951	.949	.950	.880		.951	.950	.949	.883
CFI$_u=.99$	1	8	250		.990	.990	.990	.953		.990	.989	.990	.935
			1000		.990	.990	.990	.954		.990	.990	.990	.936
		12	250		.991	.991	.990	.940		.991	.988	.990	.874
			1000		.990	.990	.990	.941		.990	.989	.990	.876
	2	8	250		.990	.987	.989	.941		.991	.988	.989	.958
			1000		.990	.989	.990	.940		.990	.989	.990	.959
		12	250		.990	.988	.990	.924		.991	.988	.989	.923
			1000		.990	.989	.990	.925		.990	.989	.990	.923
		18	250		.991	.989	.990	.915		.992	.987	.990	.862
			1000		.990	.990	.990	.915		.990	.989	.990	.863
	3	12	250		.991	.987	.989	.892		.991	.988	.989	.936
			1000		.990	.989	.990	.892		.990	.989	.990	.937
		18	250		.990	.988	.989	.877		.992	.988	.989	.883
			1000		.990	.989	.990	.878		.990	.989	.990	.883

Orig.: unadjusted CFI.

Comparing the original CFI with CFI$_u$, the former is continuously smaller. For settings in which CFI$_u=0.99$, this difference in size increases as the number of latent variables is increased, whereby CFI-values become consistently smaller with increasing model complexity. This difference is also likely connected to the different average correlation sizes within the respective conditions.

Despite the difference in average value, the corresponding unweighted and noncentrality-parameter-based indices measure very similar aspects of misspecification. This can be verified by the substantial correlations between RMSEA$$_u$$ and RMSEA (mean$$ = 0.918$$; range$$ = 0.598: 0.991$$) as well as those between CFI$$_u$$ and CFI (mean$$ = 0.913$$; range$$ = 0.553: 0.989$$) within the individual simulation conditions. Note that all indices within conditions have the same target values and, therefore, decreased estimate variances lead to smaller correlations if the sample is large and the model has many parameters.

Second, the simulation results pertaining to the ULSMV analyses for metric as well as dichotomous variables are considered. The underlying model structures were fully identical for the two variable types respectively. Adjustments were performed using approach (1.1) and (1.2).

Tables 5 and 6 give RMSEA$_u$ and RMSEA for the ULSMV analyses for each of the data settings separated for misfit type I and II respectively. Results pertaining to CFI$_u$ and CFI are contained in Table 7.

**Table 5:** Average RMSEAu and RMSEA values as well as 90%-CI coverages of ULSMV confirmatory factor models based on 1000 replications per cell. (Misfit Type I).

	$q^*$	*p*	*n*		Metric						Dichotomous				
Target					(1.1)		(1.2)		orig.		(1.1)		(1.2)		orig.
value					Est.	CIC	Est.	CIC	Est.		Est.	CIC	Est.	CIC	Est.
RMSEA$_u=.05$; CFI$_u=.95$	1	8	250		.047	.832	.048	.819	.054		.054	.691	.043	.705	.029
			1000		.050	.880	.050	.876	.059		.049	.723	.050	.863	.035
		12	250		.048	.841	.049	.825	.055		.049	.541	.047	.625	.031
			1000		.050	.872	.050	.868	.059		.049	.707	.050	.822	.035
	2	8	250		.045	.824	.048	.815	.050		.056	.700	.038	.881	.028
			1000		.050	.895	.050	.888	.055		.048	.708	.048	.856	.033
		12	250		.047	.813	.050	.786	.051		.048	.546	.042	.545	.030
			1000		.050	.889	.050	.883	.054		.049	.717	.049	.844	.034
		18	250		.047	.833	.050	.791	.048		.044	.464	.045	.409	.029
			1000		.050	.890	.050	.880	.051		.048	.720	.049	.825	.033
	3	12	250		.045	.820	.049	.784	.051		.048	.543	.039	.522	.030
			1000		.049	.894	.050	.881	.055		.048	.727	.048	.836	.034
		18	250		.047	.820	.050	.765	.048		.044	.448	.042	.344	.029
			1000		.049	.870	.050	.848	.052		.048	.687	.048	.800	.033
RMSEA$_u=.05$; CFI$_u=.99$	1	8	250		.048	.871	.050	.848	.079		.051	.703	.045	.687	.038
			1000		.050	.882	.050	.878	.083		.049	.778	.050	.880	.044
		12	250		.048	.858	.050	.829	.080		.047	.604	.049	.605	.042
			1000		.050	.886	.050	.877	.085		.049	.772	.050	.832	.045
	2	8	250		.047	.898	.050	.877	.065		.051	.700	.040	.723	.034
			1000		.050	.914	.050	.900	.068		.048	.776	.048	.887	.038
		12	250		.048	.888	.051	.835	.063		.044	.584	.043	.589	.034
			1000		.050	.908	.050	.895	.064		.047	.774	.049	.868	.037
		18	250		.047	.893	.050	.820	.057		.040	.540	.044	.362	.032
			1000		.049	.901	.050	.886	.059		.045	.739	.049	.833	.035
	3	12	250		.046	.896	.050	.808	.073		.044	.615	.041	.559	.038
			1000		.049	.909	.050	.881	.075		.047	.807	.048	.873	.040
		18	250		.047	.889	.050	.786	.065		.040	.589	.044	.292	.035
			1000		.049	.895	.050	.868	.067		.046	.789	.049	.838	.038

Est.: RMSEA estimate and CIC: $90\%$-confidence interval coverage pertaining to the respective approach; Orig. Est.: unadjusted RMSEA.

Similar to the ML analyses, RMSEA$_u$ and CFI$_u$ consistently approach their respective target values of 0.05 and 0.95/0.99 across conditions. This holds equally true for metric as well as dichotomous data. Although there are descriptive differences between approaches (1.1) and (1.2.) within singular condition, there are no notable differences in average performance.

If the sample size is $n=1000$, deviations from the true values only rarely exceed $\pm 0.001$ for metric data and $\pm 0.002$ for dichotomous data. However, if the sample size is small and if many factors are modeled based on comparably few variables, fit indices have a notable bias. In these cases, RMSEA$_u$ values are underestimated and CFI$_u$ values are overestimated. Specifically, RMSEA$_u$ underestimated its target value by up to 0.010 points and CFI$_u$ are increased by up to 0.013 points if the target value is 0.95 and 0.003 points if the target value is 0.99. Interestingly, these effects do not differ in strength between metric and dichotomous data analyses.

The deviations from the target value in small samples do most likely not result from a specific property of the new fit indices. This may be inferred from the strong differences of the original RMSEA and CFI between different sample sizes within otherwise identical conditions, which are similar in size to the target value deviations for $n=250$.

While there are no strong differences between metric and dichotomous data models with respect to average value of the new indices, differences between confidence interval coverages are more pronounced. Coverages pertaining to the metric data models are reasonably close to their target of $90\%$ if $n=1000$. For $n=250$ there are some individual conditions (misfit type II, p=18, CFI$_u=0.99$) in which CIC pertaining to approach (1.2) are very poor. This may be explained in part by the biased estimates in the respective conditions.

The CIC pertaining to the dichotomous data models perform reasonably well for approach (1.2) if $n=1000$. However, confidence intervals pertaining to approach (1.1) and, even more notable, CICs in samples with $n=250$ are too small across conditions. Again, part of this can be attributed the moderate estimation bias in small samples. Nevertheless, reliable RMSEA$_u$ confidence intervals for dichotomous data models are only obtained using approach (1.2) in larger samples.

**Table 6:** Average RMSEAu and RMSEA values as well as 90%-CI coverages of ULSMV confirmatory factor models based on 1000 replications per cell. (Misfit Type II).

	$q^*$	*p*	*n*		Metric						Dichotomous				
Target					(1.1)		(1.2)		orig.		(1.1)		(1.2)		orig.
value					Est.	CIC	Est.	CIC	Est.		Est.	CIC	Est.	CIC	Est.
RMSEA$_u=.05$; CFI$_u=.95$	1	8	250		.048	.895	.050	.872	.080		.055	.709	.044	.670	.029
			1000		.050	.920	.050	.909	.083		.049	.731	.049	.874	.034
		12	250		.048	.905	.050	.864	.081		.048	.546	.047	.662	.032
			1000		.050	.905	.050	.883	.085		.049	.752	.050	.862	.036
	2	8	250		.045	.892	.050	.830	.066		.056	.701	.036	.850	.029
			1000		.049	.905	.050	.882	.068		.048	.682	.046	.808	.034
		12	250		.046	.872	.050	.809	.063		.048	.562	.039	.548	.029
			1000		.049	.919	.050	.891	.065		.049	.722	.049	.834	.034
		18	250		.044	.868	.050	.700	.058		.044	.471	.044	.376	.030
			1000		.049	.906	.050	.852	.060		.048	.705	.049	.812	.033
	3	12	250		.043	.883	.050	.738	.073		.047	.553	.032	.396	.030
			1000		.048	.894	.050	.829	.075		.049	.689	.047	.792	.035
		18	250		.041	.876	.050	.548	.066		.043	.465	.039	.333	.029
			1000		.048	.929	.050	.814	.069		.048	.728	.048	.828	.034
RMSEA$_u=.05$; CFI$_u=.99$	1	8	250		.051	.692	.047	.694	.039		.051	.703	.045	.687	.038
			1000		.049	.780	.050	.864	.044		.049	.778	.050	.880	.044
		12	250		.047	.618	.050	.597	.042		.047	.604	.049	.605	.042
			1000		.049	.805	.050	.852	.045		.049	.772	.050	.832	.045
	2	8	250		.052	.698	.040	.790	.034		.051	.700	.040	.723	.034
			1000		.048	.780	.048	.871	.038		.048	.776	.048	.887	.038
		12	250		.044	.608	.042	.557	.034		.044	.584	.043	.589	.034
			1000		.047	.772	.049	.862	.038		.047	.774	.049	.868	.037
		18	250		.040	.574	.045	.358	.033		.040	.540	.044	.362	.032
			1000		.045	.756	.049	.832	.035		.045	.739	.049	.833	.035
	3	12	250		.045	.634	.040	.499	.037		.044	.615	.041	.559	.038
			1000		.047	.791	.048	.853	.041		.047	.807	.048	.873	.040
		18	250		.041	.687	.043	.375	.035		.040	.589	.044	.292	.035
			1000		.046	.772	.049	.823	.038		.046	.789	.049	.838	.038

Est.: RMSEA estimate and CIC: $90\%$-confidence interval coverage pertaining to the respective approach; Orig. Est.: unadjusted RMSEA.

Because dichotomous data were obtained directly from the metric data within single replications of the simulation study, correlations of fit indices based on the two types of data can be considered. Specifically, the average correlation between fit indices for metric and dichotomous data models was 0.999 for RMESA$_u$ and CFI$_u$ across conditions and adjustment approaches. Thus, not only do they approach the same target value, they are also similarly sensitive to the respective discrepancies.

As was expected, conventional noncentrality-parameter-based fit indices differ strongly between metric and dichotomous data. Specifically, RMSEA values for models based on dichotomous indicators are consistently smaller (by at least 0.020 points) than those obtained from metric variable models, which matches previous results of Monroe and Cai (2015) and Xia and Yang (2018). Also, CFI values from dichotomous data models are continuously larger than those obtained from metric data models. Thus, despite the identical model structures, fit assessments based on different variable types would lead to different conclusions using conventional fit indices. Interestingly, while RMSEA and CFI differ between metric and dichotomous data, they are virtually identical for analyses based on ML and ULSMV within corresponding conditions.

**Table 7:** Average CFI $_u$ and CFI values of ULSMV confirmatory factor models based on 1000 replications per cell.

	$q^*$	*p*	*n*		Misfit type I							Misfit type II					
Target					Metric			Dichotomous				Metric			Dichotomous		
value					(1.1)	(1.2)	Orig.	(1.1)	(1.2)	Orig.		(1.1)	(1.2)	Orig.	(1.1)	(1.2)	Orig.
CFI$_u=.95$	1	8	250		.953	.951	.902	.953	.958	.928		.954	.951	.902	.953	.958	.928
			1000		.950	.950	.903	.949	.950	.933		.950	.950	.903	.949	.951	.934
		12	250		.953	.951	.886	.949	.951	.926		.953	.950	.884	.949	.950	.925
			1000		.950	.950	.883	.950	.950	.929		.951	.950	.883	.949	.950	.928
	2	8	250		.956	.953	.918	.955	.967	.934		.957	.954	.915	.954	.963	.932
			1000		.951	.950	.918	.951	.953	.939		.953	.953	.918	.953	.954	.941
		12	250		.953	.950	.904	.952	.959	.933		.956	.951	.903	.954	.963	.934
			1000		.951	.950	.907	.951	.952	.936		.951	.950	.902	.950	.952	.934
		18	250		.955	.951	.896	.956	.957	.933		.956	.951	.892	.956	.958	.932
			1000		.951	.950	.894	.953	.952	.933		.951	.950	.889	.953	.952	.932
	3	12	250		.958	.953	.905	.953	.964	.933		.958	.952	.899	.954	.973	.934
			1000		.952	.951	.904	.951	.954	.935		.952	.951	.898	.951	.953	.933
		18	250		.956	.951	.895	.956	.960	.932		.958	.951	.891	.957	.965	.933
			1000		.951	.950	.893	.953	.953	.932		.951	.950	.886	.952	.953	.930
CFI$_u=.99$	1	8	250		.990	.990	.925	.990	.990	.980		.991	.990	.925	.990	.990	.979
			1000		.990	.990	.923	.990	.990	.980		.990	.990	.922	.990	.990	.980
		12	250		.991	.990	.888	.990	.989	.976		.991	.990	.886	.990	.989	.976
			1000		.990	.990	.878	.990	.990	.976		.990	.990	.877	.990	.990	.976
	2	8	250		.991	.990	.954	.990	.992	.981		.992	.990	.953	.990	.993	.981
			1000		.990	.990	.954	.991	.991	.983		.990	.990	.953	.990	.991	.983
		12	250		.991	.990	.936	.992	.991	.980		.991	.990	.935	.992	.992	.980
			1000		.990	.990	.937	.991	.990	.980		.990	.990	.934	.991	.990	.979
		18	250		.991	.990	.922	.993	.991	.978		.992	.990	.919	.993	.991	.977
			1000		.990	.990	.919	.992	.990	.977		.990	.990	.916	.992	.990	.976
	3	12	250		.991	.990	.919	.991	.992	.975		.993	.990	.918	.991	.993	.976
			1000		.990	.990	.918	.991	.991	.975		.991	.990	.917	.991	.991	.975
		18	250		.991	.990	.901	.992	.992	.972		.993	.990	.898	.992	.992	.972
			1000		.990	.990	.900	.991	.990	.970		.991	.990	.894	.991	.990	.970

Orig.: unadjusted CFI.

Deriving cutoff values for RMSEA$_u$ and CFI$_u$ by comparing them to their traditional counterparts is only possible to a limited extent, because of their non-constant relation across simulation conditions. Particularly if the target value of CFI$_u$ is 0.99, this non-constant relation is most pronounced. Nevertheless, because RMSEA$_u$ does not differ strongly from the RMSEA, although the former yields somewhat smaller values, similar cutoff conventions, as suggested by Hu and Bentler (1999), may be viable. For CFI$_u$, which always exceeds the CFI, a value of 0.99 may relate sufficiently to an CFI value of 0.95. However, it may be more advisable to refine cutoff criteria based on empirical experience, similar to the initial determination of cutoffs pertaining to RMSEA and CFI, rather than to restrict to relying on an analogy that only partly applies.

Another perspective for identifying a suitable cut-off value may be to simulate metric data with predefined target values for the original RMSEA and CFI. In this way, cut-off values for the new indices can be specifically linked to their established counterparts. A table containing the expected values of the new fit indices based on target values on RMSEA $=0.05$ and CFI $=0.05$ (for present simulation conditions) is contained in the supplementary materials of this article.

## Discussion

In the present article, unweighted approximation error estimates have been developed to resolve the scaling related limitations of noncentrality-parameter-based fit assessments. These unweighted estimates were inserted as replacements of conventional noncentrality parameter estimates in the established fit indices RMSEA and CFI. As was demonstrated, the modified indices have a simple interpretation with respect to correlation residuals due to systematic error. In addition, the new indices yield identical values for latent variable models based on metric *and* dichotomous variables. Clearly, these two properties make them viable alternatives or additions to their established counterparts. Moreover, the impact of different discrepancy functions on typical fit index values, which has recently been reported by Shi and Maydeu-Olivares (2020), does not affect the new indices, because any scaling information specific to parameter estimation is eliminated.

The finite sample performance of the new fit indices was evaluated using simulation studies. Metric data was analyzed using ML and ULSMV, whereas analyses of dichotomous data were only performed using ULSMV. Of course other estimators could have been used for model fitting. For instance, it would have been similarly viable to analyze categorical data with ML or any other least square discrepancy. In future studies it might be interesting to investigate a more comprehensive selection of model estimation approaches.

For all simulation settings, the new fit indices (*i*) closely and consistently approached their target values and (*ii*) yielded virtually identical values for metric and dichotomous variables. For metric data models in combination with ML estimation, the match between fit index estimate and target value may be considered as completely satisfactory to recommend them for empirical applications already for sample size of $n=250$. When using ULSMV, the small sample performance was limited in some conditions. However, for a sample size of $n=1000$, fit index estimates were similarly accurate on average for analyses based on metric as well as dichotomous data.

Comparing the different approaches for obtaining a point estimate of the unweighted approximation error, there were only marginal and mostly nonsystematic differences. In conditions with ML estimation, approaches (1.1) and (2) had a slight tendency to overestimate and approach (1.2) to underestimate the indices. For ULSMV estimation with dichotomous data, estimates based on approach (1.1) were on average (but not across all conditions) slightly more accurate. Overall, for obtaining a point estimate, all approach may be considered as equally suited.

With respect to confidence interval coverage, analyses based on metric data yielded satisfactory results, with some exceptions. Particularly ML estimation in combination with fit indices based on approaches (1.1) and (1.2) worked generally well, whereas confidence intervals for approach (2) were slightly too narrow on average. For ULSMV analyses with metric data, approaches (1.1) and (1.2) work comparably well. However, for misfit type II and $q^* \ge 2$ and $p \ge 12$ there was a surprising decline in CIC.

For dichotomous data analyses, confidence intervals were uniformly too narrow. While for $n=1000$ the impact may be considered as moderate to small, for smaller samples and complex models, coverages were poor. In such cases it might be advisable to consider implementing a bootstrapping approach (e.g., Zhang & Savalei, 2016).

Clearly, while the indices as well as CIC appear to consistently approach their target values, there is some bias in small samples in combination with complex analyses. However, this is not specific to the new indices, which can be concluded from the identical deviations of RMSEA and CFI depending on sample size. In addition, similar results pertaining to CIC are also typical (e.g., Savalei, 2021; Zhang & Savalei, 2016).

The partially worse performance of fit indices in connection with ULSMV estimation is not entirely unexpected. One reason for this might be the limited adherence of the ULSMV test statistic to a noncentral $\chi ^2$-distribution under misspecification. In general, least-square based discrepancy functions have shown to perform slightly worse under some conditions than ML (e.g., Olsson et al. 2004). Also, the ULSMV-adjustment might have limitations under some aspects of model misspecification, because it is based on theorems of Box (1954) on the sum of least squares, which assume deviations to have expectations of zero. However, for now these considerations are mere conjectures.

For the established, noncentrality-parameter-based versions of RMSEA and CFI, results of Monroe and Cai (2015) and Xia and Yang (2018) were replicated, demonstrating that these fit indices yield different conclusions depending on the variables’ level-of-measurement for identical model structures. Specifically, RMSEA and CFI based on dichotomous data models continuously suggested a better model fit than those pertaining to metric data models. Thus, the universality of cutoff values pertaining to conventional fit indices is questionable.

Because of the different scaling, RMSEA and RMSEA$_u$ as well as CFI and CFI$_u$ yielded different values on average as well as across simulation study conditions. Because the conventional noncentrality parameter weighs the same absolute residuals more strongly for comparably larger correlations, the RMSEA becomes larger relative to RMSEA$_u$ as the average correlation size increases. Whether this property may be considered as an advantage or disadvantage is not obvious. While from the perspective of random deviations, identical absolute differences in larger parameters are more meaningful, this does not necessarily mean that the same perspective is sensible for considering systematic deviations.

With respect to sensitivity to detect misfit, the consistently large correlations between RMSEA and CFI with the corresponding new indices RMSEA$_u$ and CFI$_u$ suggest that the latter differentiate similarly well. Consequently, it appears natural that discriminability results of previous large-scale fit index comparisons (e.g., Hu & Bentler, 1998; 1999) should similarly apply to RMSEA$_u$ and CFI$_u$.

Although the primary objective of the present article is the introduction of the unweighted approximation error estimate as a suitable basis for fit index development, rather than a comprehensive evaluation of new fit indices, potential cutoff values for RMSEA$_u$ and CFI$_u$ may already be considered. While comparing the new and the original indices did not provide a simple rationale which cutoff values should be preferred, at least some conclusions may be drawn. As was outlined at the end of the results section, a sensible cutoff value for RMSEA$_u$ might be similar to that of the original RMSEA, that is, 0.05 for models with a good fit. For CFI$_u$ a cutoff value of 0.99 appears recommendable, because of its match to a CFI of 0.95 for models with smaller numbers of variables. However, the results also suggest that it might be useful to consider both new indices simultaneously. Specifically, while in case of CFI$_u = 0.95$, RMSEA$_u = 0,05$ appears to have a close relation to a similarly sized RMSEA, a slightly decreased RMSEA$_u$-value would be expected in cases of CFI$_u = 0.99$.

While, I consider the present cutoff recommendations as sufficient for now, they need to be considered as preliminary. Because the new indices are intended as measures in their own right, having their own specific interpretations, more extensive evaluations, such as those conducted by Hu and Bentler (1998) and Hu and Bentler (1999), would certainly be a sensible (or even necessary) pursuit of future research.

## Supplementary Information

Below is the link to the electronic supplementary material.

Supplementary file 1 (docx 16 KB)

Supplementary file 2 (pdf 134 KB)

Supplementary file 3 (txt 21 KB)

## Footnotes

1 For the tetrachoric correlation, we assume that variables have been dichotomized at their median.

2 It would have been also viable to analyze the dichotomized data using ML. The present option have been selected based on conceptual proximity and a compromise of relevance and conciseness.
