# Supplementary material for: Advantages of Using Unweighted Approximation Error Measures for Model Fit Assessment
Source: Psychometrika. 2023 Apr 18;88(2):413–33. doi: 10.1007/s11336-023-09909-6 (PMC10188575; doi:10.1007/s11336-023-09909-6)
Supplement: Supplementary file 1 — (docx 16 KB) [file 11336_2023_9909_MOESM1_ESM.docx]

$\mathrm{RMSEA}_{u}$ simulation estimates for a true RMSEA value of 0.05 (and CFI = 0.95)

|  |  |  | Misfit Type I | | | | Misfit Type II | | | |
| --- | --- | --- | --- | --- | --- | --- | --- | --- | --- | --- |
| n | p | q* | (1.1) | (1.2) | (2) | RMSEA | (1.1) | (1.2) | (2) | RMSEA |
| 250 | 8 | 1 | 0.032 | 0.034 | 0.038 | 0.045 | 0.038 | 0.039 | 0.040 | 0.047 |
| 1000 | 8 | 1 | 0.037 | 0.037 | 0.037 | 0.049 | 0.041 | 0.041 | 0.040 | 0.050 |
| 250 | 12 | 1 | 0.029 | 0.032 | 0.033 | 0.049 | 0.037 | 0.039 | 0.036 | 0.049 |
| 1000 | 12 | 1 | 0.032 | 0.033 | 0.033 | 0.050 | 0.040 | 0.040 | 0.038 | 0.050 |
| 250 | 8 | 2 | 0.039 | 0.042 | 0.043 | 0.046 | 0.037 | 0.038 | 0.040 | 0.046 |
| 1000 | 8 | 2 | 0.042 | 0.043 | 0.042 | 0.049 | 0.040 | 0.041 | 0.040 | 0.049 |
| 250 | 12 | 2 | 0.034 | 0.038 | 0.038 | 0.049 | 0.036 | 0.038 | 0.036 | 0.050 |
| 1000 | 12 | 2 | 0.037 | 0.038 | 0.038 | 0.050 | 0.038 | 0.038 | 0.037 | 0.050 |
| 250 | 18 | 2 | 0.030 | 0.034 | 0.036 | 0.051 | 0.033 | 0.036 | 0.033 | 0.051 |
| 1000 | 18 | 2 | 0.033 | 0.033 | 0.034 | 0.050 | 0.036 | 0.036 | 0.034 | 0.050 |
| 250 | 12 | 3 | 0.035 | 0.041 | 0.039 | 0.049 | 0.035 | 0.038 | 0.036 | 0.049 |
| 1000 | 12 | 3 | 0.040 | 0.041 | 0.040 | 0.050 | 0.038 | 0.038 | 0.037 | 0.050 |
| 250 | 18 | 3 | 0.031 | 0.036 | 0.037 | 0.051 | 0.032 | 0.035 | 0.033 | 0.051 |
| 1000 | 18 | 3 | 0.034 | 0.035 | 0.035 | 0.050 | 0.035 | 0.036 | 0.034 | 0.050 |

$\mathrm{CFI}_{u}$ simulation estimates for a true CFI value of 0.95 (and RMSEA = 0.05)

|  |  |  | Misfit Type I | | | | Misfit Type II | | | |
| --- | --- | --- | --- | --- | --- | --- | --- | --- | --- | --- |
| n | p | q* | (1.1) | (1.2) | (2) | CFI | (1.1) | (1.2) | (2) | CFI |
| 250 | 8 | 1 | 0.985 | 0.984 | 0.982 | 0.949 | 0.981 | 0.979 | 0.979 | 0.946 |
| 1000 | 8 | 1 | 0.984 | 0.984 | 0.983 | 0.950 | 0.980 | 0.980 | 0.980 | 0.949 |
| 250 | 12 | 1 | 0.992 | 0.991 | 0.989 | 0.948 | 0.987 | 0.986 | 0.987 | 0.948 |
| 1000 | 12 | 1 | 0.991 | 0.991 | 0.990 | 0.950 | 0.987 | 0.986 | 0.987 | 0.950 |
| 250 | 8 | 2 | 0.975 | 0.972 | 0.972 | 0.948 | 0.982 | 0.980 | 0.979 | 0.948 |
| 1000 | 8 | 2 | 0.974 | 0.973 | 0.973 | 0.950 | 0.981 | 0.981 | 0.981 | 0.950 |
| 250 | 12 | 2 | 0.985 | 0.983 | 0.981 | 0.949 | 0.988 | 0.987 | 0.987 | 0.947 |
| 1000 | 12 | 2 | 0.984 | 0.983 | 0.983 | 0.950 | 0.988 | 0.987 | 0.988 | 0.950 |
| 250 | 18 | 2 | 0.992 | 0.990 | 0.988 | 0.948 | 0.993 | 0.992 | 0.993 | 0.947 |
| 1000 | 18 | 2 | 0.991 | 0.990 | 0.990 | 0.950 | 0.993 | 0.993 | 0.993 | 0.950 |
| 250 | 12 | 3 | 0.982 | 0.978 | 0.978 | 0.949 | 0.988 | 0.987 | 0.987 | 0.948 |
| 1000 | 12 | 3 | 0.980 | 0.979 | 0.979 | 0.949 | 0.987 | 0.987 | 0.987 | 0.950 |
| 250 | 18 | 3 | 0.989 | 0.986 | 0.985 | 0.947 | 0.994 | 0.993 | 0.993 | 0.947 |
| 1000 | 18 | 3 | 0.988 | 0.988 | 0.987 | 0.950 | 0.993 | 0.993 | 0.993 | 0.950 |
