# Supplementary material for: Advantages of Using Unweighted Approximation Error Measures for Model Fit Assessment
Source: Psychometrika. 2023 Apr 18;88(2):413–33. doi: 10.1007/s11336-023-09909-6 (PMC10188575; doi:10.1007/s11336-023-09909-6)
Supplement: Supplementary file 2 — (pdf 134 KB) [file 11336_2023_9909_MOESM2_ESM.pdf]

```
1   #~~~~~~~~~~~~~~~#
2   # INSTRUCTIONS #
3   #~~~~~~~~~~~~~~~#
4   # The following code can be used for calculating RMSEA_u and CFI_u
5   # using approaches (1.1), (1.2), and (2). It can be used for uni- and
6   # multidimensional CFA models. For a more flexible use, the Ffun-
7   # function (lines 24 to 31) needs to be adapted.
8   # An example data is provided with this article, which contains four
9   # metric variables. Its location needs to be specified in line 37.
10  # The corresponding parameter estimates (standardized ML loadings)
11  # are entered in line 54. Estimate vectors of multidimensional CFA
12  # models first contain all loadings and then the factor correlations.
13
14  library(matrixcalc)
15  library(numDeriv)
16
17  #~~~~~~~~~~~~~~~~~#
18  # basic functions #
19  #~~~~~~~~~~~~~~~~~#
20  #matrix trace
21  tr <- function(x) sum(diag(x))
22
23  #model implied correlations based on loadings and factor correlations
24  Ffun <- function(x) {
25    LAM <- diag(q) %x% rep(1,p/q)
26    LAM[LAM==1] <- x[(q2+1):(q2+p)]
27    PHI <- diag(q)/2
28    PHI[lower.tri(PHI)] <- x[1:q2]
29    PHI <- PHI + t(PHI)
30    (LAM %*% PHI %*% t(LAM))[lower.tri(diag(p))]
31  }
32
33  #~~~~~~~#
34  # data #
35  #~~~~~~~#
36  #!!! specify data location !!!
37  X <- read.table('...\\example_data.txt')
38
39  #settings
40  n  <- nrow(X)
41  p  <- ncol(X)
42  p2 <- (p*(p-1))/2
43  q  <- 1
44  q2 <- (q*(q-1))/2
45  df <- p2 - p - q2
46
47  #sample correlations
48  S0 <- cor(X)
49
50  #~~~~~~~~~~~~~~~~#
51  # ML CFA-results #
52  #~~~~~~~~~~~~~~~~#
53  #std. loading estimates
54  est <- c(0.3177388, -0.3547450,  0.2909282, -0.4468409)
55
56  #model implied correlations
57  s1 <- Ffun(est)
58  S. <- diag(p)/2
59  S.[lower.tri(S.)] <- s1
60  S1 <- S. + t(S.)
61
62  #ML test statistic
63  T_ML <- n * (log(det(S1)) + tr(S0 %*% solve(S1)) - log(det(S0)) - p)
64
65  #ML test statistic (base model)
66  T_B_ML <- n * (tr(S0 %*% diag(p)) - log(det(S0)) - p)
67
68  #~~~~~~~~~~~~~~~~~#
69  # ULS discrepancy #
70  #~~~~~~~~~~~~~~~~~#
71  #correlation residuals
72  e     <- (S0-S1)[lower.tri(S0)]
73
```

```r
#model discrepancy
F_uls <- sum(e^2)

#base model discrepancy
F_B_u <- sum(S0[lower.tri(S0)]^2)

#~~~~~~~~~~~~~~~~~~~~#
# calculate matrices #
#~~~~~~~~~~~~~~~~~~~~#
#I
I   <- diag((p*(p-1))/2)

#Gamma
X2 <- X^2
C  <- combn(p,2)
for(i in 1:ncol(C)) X2 <- cbind(X2, X[,C[1,i]]*X[,C[2,i]])
GAM <- var(X2)

#W
Dp  <- duplication.matrix(p)
D.  <- svd.inverse(Dp)
cr  <- which(rowSums(D.^2) != 1)
W.  <- 2 * D. %*% (S0 %x% S0) %*% t(D.)
W   <- solve(W.)[cr,cr]

#Delta
DEL <- jacobian(Ffun, est)

#~~~~~~~~~~~~~~~~#
# approach (1.1) #
#~~~~~~~~~~~~~~~~#
U_1.1  <- I - DEL %*% solve(t(DEL) %*% DEL) %*% t(DEL)
V_1.1  <- (U_1.1 %*% GAM[cr,cr])/n
tr_1.1 <- tr(V_1.1)
s2_1.1 <- 2 * tr(V_1.1^2) + 4 * t(e) %*% V_1.1 %*% e

#corrected RMSEA_u estimate
k_1.1   <- 1 - (s2_1.1) / (4*F_uls^2)
R_u_1.1 <- 1/k_1.1 * sqrt(max(c(F_uls - tr_1.1, 0))/df)

#RMSEA_u standard error
SE_1.1  <- sqrt(s2_1.1/(k_1.1^2*4*F_uls))[1]

#CFI_u
C_u_1.1 <- 1 - (F_uls-tr_1.1) / (F_B_u-tr_1.1)

#output
round(
c(RMSEA_u_1.1 = R_u_1.1,
  low.CI      = max(0, R_u_1.1-qnorm(.95)*SE_1.1),
  upp.CI      = R_u_1.1+qnorm(.95)*SE_1.1,
  CFI_u_1.1   = C_u_1.1), 3)

#~~~~~~~~~~~~~~~~#
# approach (1.2) #
#~~~~~~~~~~~~~~~~#
U_1.2  <- I - DEL %*% solve(t(DEL) %*% W %*% DEL) %*% t(DEL) %*% W
V_1.2  <- 1/n * (U_1.2) %*% GAM[cr,cr] %*% t(U_1.2)
tr_1.2 <- tr(V_1.2)
s2_1.2 <- 2 * tr(V_1.2^2) + 4 * t(e) %*% V_1.2 %*% e

#corrected RMSEA_u estimate
k_1.2   <- 1 - (s2_1.2) / (4*F_uls^2)
R_u_1.2 <- 1/k_1.2 * sqrt(max(c(F_uls - tr_1.2, 0))/df)

#RMSEA_u standard error
SE_1.2  <- sqrt(s2_1.2/(k_1.2^2*4*df*F_uls))[1]

#CFI_u
C_u_1.2 <- 1 - (F_uls-tr_1.2) / (F_B_u-tr_1.2)

#output
round(
```

```
c(RMSEA_u_1.2 = R_u_1.2,
  low.CI      = max(0, R_u_1.2-qnorm(.95)*SE_1.2),
  upp.CI      = R_u_1.2+qnorm(.95)*SE_1.2,
  CFI_u_1.2   = C_u_1.2), 3)

#~~~~~~~~~~~~~~#
# approach (2) #
#~~~~~~~~~~~~~~#
s2_2  <- s2_1.2
#model ADR
ADR   <- (T_ML - df) / T_ML
#base model ADR (for CFI_u)
ADR_B <- (T_B_ML - p2) / T_B_ML

#corrected RMSEA_u estimate
k_2   <- 1 - (s2_2)/(8*F_uls^2)
R_u_2 <- 1/k_2 * sqrt(max(c(ADR * F_uls, 0))/df)

#RMSEA_u standard error
SE_2  <- sqrt((s2_2*ADR)/(k_2^2*4*df*F_uls))[1]

#CFI_u
C_u_2 <- 1 - (ADR * F_uls) / (ADR_B * F_B_u)

#output
round(
c(RMSEA_u_2 = R_u_2,
  low.CI    = max(0, R_u_2-qnorm(.95)*SE_2),
  upp.CI    = R_u_2+qnorm(.95)*SE_2,
  CFI_u_2   = C_u_2),3)
```
